# Enhanced all-optical modulation in a graphene-coated fibre with low insertion loss

**DOI:** 10.1038/srep23512

**Published:** 2016-03-22

**Authors:** Haojie Zhang, Noel Healy, Li Shen, Chung Che Huang, Daniel W. Hewak, Anna C. Peacock

**Affiliations:** 1 Optoelectronics Research Centre, University of Southampton, Highfield, Southampton SO17 1BJ, UK; 2 Emerging Technology and Materials Group, School of Electrical and Electronic Engineering, Newcastle University, Newcastle upon Tyne NE1 7RU, UK

## Abstract

Graphene is a highly versatile two-dimensional material platform that offers exceptional optical and electrical properties. Of these, its dynamic conductivity and low effective carrier mass are of particular interest for optoelectronic applications as they underpin the material’s broadband nonlinear optical absorption and ultra-fast carrier mobility, respectively. In this paper, we utilize these phenomena to demonstrate a high-speed, in-fibre optical modulator developed on a side-polished optical fibre platform. An especially low insertion loss (<1 dB) was achieved by polishing the fibre to a near atomically smooth surface (<1 nm RMS), which minimized scattering and ensured excellent contact between the graphene film and the fibre. In order to enhance the light-matter interaction, the graphene film is coated with a high index polyvinyl butyral layer, which has the added advantage of acting as a barrier to the surrounding environment. Using this innovative approach, we have fabricated a robust and stable all-fibre device with an extinction ratio as high as 9 dB and operation bandwidth of 0.5 THz. These results represent a key step towards the integration of low-dimensional materials within standard telecoms networks.

Graphene is the most prominent of an emerging catalogue of two-dimensional materials that are currently spear-heading intense research across the optoelectronics community^1^. Formed by just a single layer of carbon atoms, graphene’s unique linear and gapless electronic band structure gives rise to some remarkable properties such as high electron mobility, high nonlinearity, and a strong uniform absorption of 2.3% that extends across the near-infrared wavelength region^2^,^3^,^4^. To date, these properties have been exploited for the development of a wide range of ultra-broadband graphene-based devices including polarizers^5^, photodetectors^6^, optical limiters^7^,^8^, and wavelength convertors^9^. Furthermore, it is also possible to engineer additional optoelectronic functionality into the devices by electrically or chemically tuning graphene’s Fermi level. For example, electrically driven graphene-based absorption modulators have been demonstrated, albeit at speeds limited by the driving circuitry^10^,^11^.

An alternative approach that offers a straightforward route to ultra-fast modulation is to exploit the strong saturation of graphene’s absorption due to Pauli blocking, whereby the generated carriers fill up the valence bands preventing any further transitions. By employing a pump-probe scheme, all-optical modulation has recently been demonstrated in a graphene-coated microfibre device with an operation speed approaching 200 GHz^12^. Although these results represent a nice first demonstration, the interaction between the evanescent field of the 1 μm diameter fibre and the graphene coating was quite modest, limiting the modulation depth to 2 dB. Thus this experiment highlights the importance of achieving a strong light-matter overlap to realize high performance optoelectronic devices using two-dimensional material systems^10^,^13^. Addressing this issue is becoming an increasingly important and timely topic of research, with several approaches already being pursued^13^,^14^.

In this paper, we present a robust, all-optical modulator with a large 9 dB extinction ratio and an operation bandwidth of 0.5 THz. The device is built into a side-polished window of a standard optical fibre and is thus immediately compatible with existing fibre networks. By retaining a small cladding buffer between the core and the polished window, the insertion loss of the device is less than 1 dB, which represents a record low value for this type of device^5^. The light-matter interaction with the graphene film is then enhanced via the addition of a high index polyvinyl butyral (PVB) over-layer, which draws the evanescent tail of the propagating mode out of the core. The interaction of light with low-dimensional materials is notoriously difficult and until now, the level of interaction required for practical non-linear devices, of varying geometries, has been accompanied with unacceptable losses^15^,^16^,^17^. Compared to these previous devices, our approach presents a significant improvement in performance in all key measures to simultaneously obtain low loss, high speed, and high modulation depth. This is a significant advancement that will open the door to the practical integration of two-dimensional materials with traditional optical fibres.

## Results

### Device design and fabrication

**Figure 1:**
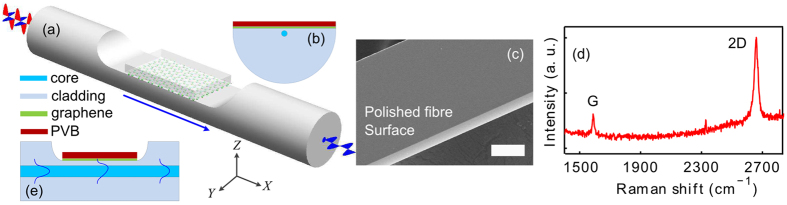
Graphene modulator design. (**a**) Schematic illustration of the graphene modulator, the interaction length is ~5 mm. (**b**) Cross-sectional view of the device. (**c**) SEM image of the polished fibre, showing ultra-smooth surface. (**d**) Raman spectrum of a typical graphene sheet, with 2D:G ratio of 4:1 and a 2D peak FWHM of 25 cm^−1^. (**e**) A schematic of the longitudinal cross-section of the modulator, solid blue lines indicate the propagating electromagnetic field.

Schematic representations of our graphene-coated modulator are illustrated in Fig. 1(a,b & e). The device is developed on a side-polished fibre so that the core guided mode can interact with the graphene film without breaking its path. The polishing process was developed to produce adiabatic transitions from the fibre’s full circular geometry to the uniform D-shaped region, which forms the interaction window (Methods and Supplementary Information SF1). A typical window length for our devices is ~1 cm and, as previously mentioned, a cladding buffer of 1 μm was retained across this region. The result is a polished fibre with negligible transmission loss, where the exposed surface provides lateral access to the light that propagates in the fibre. The potential strength of the light-material interaction was determined by dropping a high index liquid onto the polished surface and measuring the change in transmission. Using this method, it was determined that the uniform polished region permits access to more than 30 dB of the propagating light. Our polishing process is based on a standard procedure using a custom-made polishing block, which yields a uniform interaction region and an ultra-smooth surface with a roughness of <1 nm RMS (See Fig. 1c and Supplementary Information SF1). Smooth surfaces are required to reduce scattering losses that are amplified when high index materials are brought into contact with the polished surface. The graphene mono-layer was prepared via a chemical vapor deposition (CVD) method^18^ (Methods and Supplementary Information SF2), and spin-coated with a 1 μm thick layer of PVB before being transferred onto the polished region of the fibre (Supplementary Information SF3). Care was taken to ensure that a graphene layer of length 5 mm was in direct contact with the polished surface, following which the device was baked at 60 °C for 10 mins to improve the film-fibre adhesion. Raman spectroscopy was used to determine the quality of the graphene mono-layer after the transfer process was complete (see Methods). A typical spectrum is shown in Fig. 1d, from which the mono-layer nature of our film is confirmed by the 4:1 ratio of the peak intensities (2D and G) and the 25 cm^−1^ full width half maximum (FWHM) of the 2D peak^19^. For mono-layer graphene prepared via the CVD method, the material is often doped due to the substrate material and the chemical potential is typically on the order of 0.1 eV^20^. For this potential, the TM mode of the polished fibre interacts preferentially with the graphene layer^21^. Our device takes advantage of the polarization dependence as the incident light can be aligned to efficiently couple into the graphene layer to be absorbed, thereby maximizing the achievable modulation for a specific pump power.

**Figure 2:**
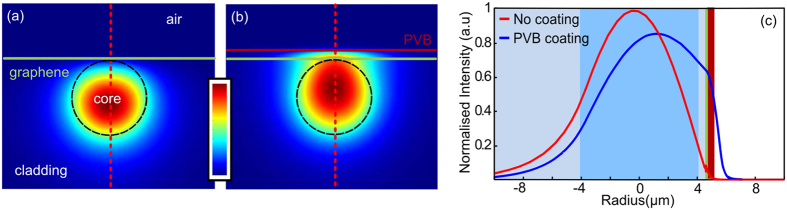
Numerically calculated modes of the side-polished device. (**a**) Fundamental fibre mode with no over-layer. (**b**) Mode with a 1 μm thick PVB layer at the polished surface. (**c**) Normalized cross-sessional line scan along dashed red lines indicated in (**a,b**).

To illustrate the principle of the enhanced light-matter interaction, the longitudinal cross-section of the device is shown in Fig. 1e. For an operation wavelength of 1550 nm, as used in our experiments, the PVB over-layer has a refractive index of n = 1.48, which is slightly higher than that of the silica fibre cladding (n = 1.44) and so draws the evanescent tail of the propagating mode out of the core. To quantify the level of overlap with and without the PVB over-layer, finite element modelling of the device mode properties was conducted (see Methods). Figure 2a shows the intensity distribution of the TM modes in the side-polished section coated with (a) graphene-only and (b) graphene-PVB, clearly illustrating the influence of the high index layer. By plotting a cross-sectional view of the mode profiles in Fig. 2c, we can estimate an increase in the interaction between the mode and the graphene film of >10 dB due to the PVB coating. It is worth pointing out that because the PVB is only 1 μm thick, the propagating mode in this device is still bound to the core, i.e., the PVB layer does not induce any radiation loss.

### Characterisation – linear regime

We start our investigations of the transmission properties of the graphene-PVB device by comparing the losses of the TM and TE modes using a polarized continuous wave (CW) laser at 1550 nm. The input power was set to 0 dBm and the polarization was tuned between the modes using a half wave plate. When coupling into the TE mode of the device, the reduction in output power was <1 dB, which is commensurate with insertion losses of commercially available fibre devices. The experiment was repeated again for the TM mode and the power was at a minimum of −31 dBm, as expected for our CVD-produced graphene film. This large, 29 dB attenuation difference between the TM and TE modes provides a good indication of the high purity of our graphene sheet and the strong light-matter interaction that has been achieved. We note that the PVB material used here is largely transparent across the transmission window of the silica fibre and thus the device should be operable across the entire telecom band. To test the broadband nature of the device, additional transmission measurements were undertaken at wavelengths spanning 1425–1600 nm, from which it was confirmed that the extinction ratio did not drop below 25 dB in this region (see Supplementary Information SF4).

### Characterisation – nonlinear regime

**Figure 3:**
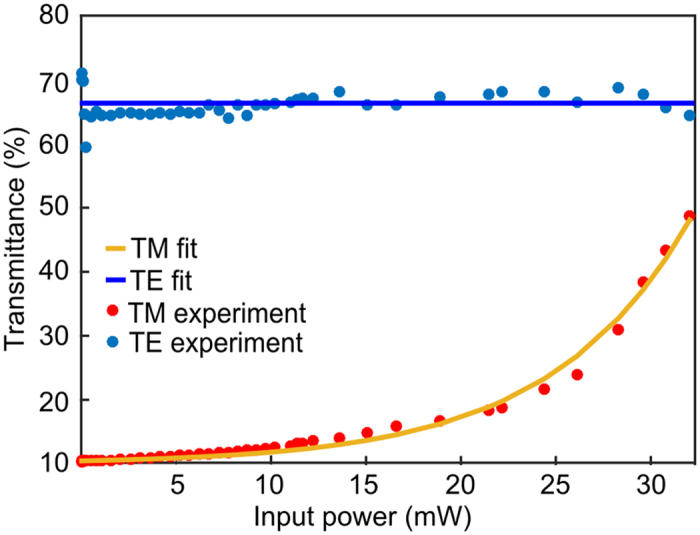
Non-linear absorption saturation. Transmittance measurements of graphene coated fibre device as a function of power.

Characterization of the nonlinear saturable absorption induced by Pauli blocking was then undertaken using a high power fibre laser operating at 1540 nm, with a duration of 750 fs (FWHM) and a repetition rate of 40 MHz. Figure 3 shows the transmittance of the TE and TM modes as a function of increasing average power coupled into the device. This clearly shows that up to P_ave_~10 mW the absorption of the TM mode is linear, however, for higher input powers the absorption becomes nonlinear and the transmitted light starts to increase exponentially, eventually reaching a transmittance of 60% for P_ave_~32 mW. In contrast, the transmission of the TE mode remains linear when the input power is increased up to the maximum available power. This is due to the minimal interaction between the TE mode and the graphene sheet; a phenomenon that forms the basis of the graphene polarizer^5^. From these results, we can deduce the maximum extinction ratio for all-optical modulation using this device to be 22 dB. However, it is anticipated that this could be improved with stronger optical pumping.

**Figure 4:**
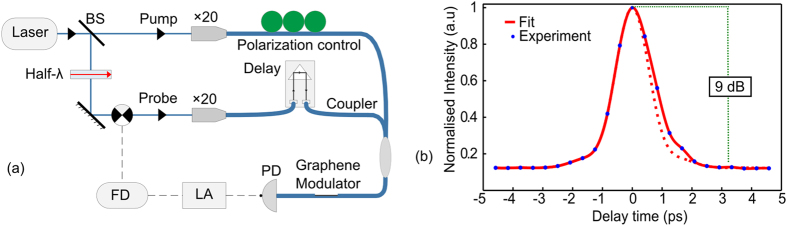
Measurement of temporal dynamics. (**a**) A schematic illustration of the experimental set-up used for high intensity optical characterization. BS: beam splitter; FD: frequency driver; LA: lock-in amplifier; PD: photodiode. (**b**) Temporally resolved speed of the all-optical modulator. Fitted curve (red line). The dashed red line is mirroring of the rising edge of the pulse and is included to highlight the slower falling edge which is due to the carrier recovery in the graphene sheet.

In order to determine the temporal dynamics of the saturable absorption we employed a simple pump-probe modulation scheme, as illustrated in Fig. 4a. In this set-up, the 1540 nm fibre laser was split into a high power pump (P_ave_~20 mW, corresponding to a peak power of P_p_~720 W) and a weak probe component (P_ave_~130 μW, P_p_~4 W). The probe was modulated at 100 Hz using an optical chopper connected to a lock-in amplifier to discriminate between the two signals. Polarization controllers were used to ensure that both the pump and probe were aligned to couple into the TM surface wave, and a 10 ps adjustable delay was inserted into the probe’s path to control the overlap between the two pulses. The pump and probe pulses were then recombined using a fused tapered coupler before being connected to the input arm of the device under test. Figure 4b shows that the power of the transmitted probe signal can be modulated with an extinction ratio of~9 dB, over a timescale of 2 ps as the pump-probe overlap is tuned. Overlaying the measured response with that of the pump itself, it is clear that the rise time is simply governed by the pump duration, however the fall time is slightly longer, which can be attributed to the intrinsic carrier recovery processes in graphene. These relaxation processes occur on two different timescales, which we estimate from the response to be τ_1_~0.1 ps and τ_2_~0.4 ps, in good agreement with previous reports^22^. Interestingly, τ_2_ has been found to be directly proportional to the crystal coherence length in the graphene sheet and thus a possible route to increasing the modulator’s intrinsic speed would be to engineer a level of disorder into the lattice.

## Discussion

In conclusion, we have designed and experimentally demonstrated a graphene-based all-optical modulator with a high extinction ratio of 9 dB and a speed of 0.5 THz. When compared to previous devices, our method results in an enhanced interaction with the graphene sheet to produce a significant increase in modulation depth, while at the same time ensuring a large decrease in insertion loss. This is best illustrated by applying the figure of merit (FOM) proposed in ref. 23, defined as the extinction ratio divided by the insertion loss, which reveals our device to have a FOM = 9, almost double that of the current state of the art^23^. As the device is built into a side-polished optical fibre it is also extremely robust and stable, and thus represents the first practical graphene-based optical fibre component. Furthermore, it can be packaged using methods that are well-established for existing fibre devices, enabling immediate compatibility with standard infrastructures. This method for enhancing light-matter interactions could be easily transferred to other two-dimensional materials to realize functionalized side-polished fibre devices that are capable of generating, modulating, and detecting light in next generation all-fibre optoelectronic systems.

## Methods

### Fibre polishing

Standard Corning SMF-28 step-index single-mode fibres with 125 μm diameter cladding, 8.2 μm diameter core, and 0.14 numerical aperture are used for the construction of the devices. An aluminium block was prepared with large-radius curved-sides, a 1 cm long flat-centre and U-shape cross-section groove cut down the middle. The radius of the two curved-side sections was fixed at 30 cm to form an adiabatic transition region and the depth of the groove was ~65 μm to allow polishing to the desired depth. A 3 cm long section of the fibre was stripped of its buffer jacket and bonded into the groove with a wax. The bulk of the top section of the fibre was removed using 3 μm aluminium oxide film, following which it was polished to a surface quality of~1 nm RMS using a proprietary polishing film (UltraTec).

### Graphene CVD

A typical CVD system for the fabrication of graphene is shown in the Supplementary Information SF2. The substrate used for the graphene fabrication is Cu foil (99.8% purity from Alfa Aesar). A 25 μm thick Cu foil substrate was cleaned by acetone, isopropanol and followed by acetic acid to remove native oxide. The cleaned Cu foil was dried with nitrogen gas and then loaded into the CVD system. The CVD reaction chamber (50 mm OD quartz tube) was evacuated to 2 mBar, and then backfilled to ambient pressure with 6% H_2_/Ar gas. After that, the temperature was increased to 1000 °C with 6% H_2_/Ar gas flow of 100 sccm and the chamber pressure maintained at 20 mBar. The Cu foil was annealed at these conditions for 30 min. The graphene growth was performed by flowing 5% CH_4_/Ar together with 6% H_2_/Ar gases for 20 min with flow rates of 100 sccm and 210 sccm, respectively. Finally, the CH_4_/Ar flow was turned off to finish the graphene deposition and the Cu foil was cooled down naturally.

### Materials Characterization

The as-grown mono-layer graphene has dimensions of 80 mm × 90 mm, as shown in Supplementary Information SF5. The quality of this graphene on Cu foil substrate was characterized by a Renishaw RamaScope equipped with a CCD camera. A 633 nm laser was used to excite the sample and the Raman spectrum was measured from 1250 cm^−1^ to 2850 cm^−1^ with a resolution of 0.1 cm^−1^. The Raman features of mono-layer graphene are the G peak at ~1580 cm^−1^ and a symmetric 2D peak at ~2700 cm^−1^ with full-width half maximum (FWHM) of~25 cm^−1^. The negligible D peak, normally seen in the spectrum near ~1350 cm^−1^, indicates that high quality (i.e., low defect level) graphene has been produced. In general, the appearance of the D peak indicates the disorder in the carbon lattice such as the domain boundaries, lattice defects or distortion. In addition to the shape of the 2D peak, it is also known that the ratio of I2D/IG can be used to determine the number of graphene layers. The typical I2D/IG ratios of single layer and bilayer exfoliated graphene are ~2–3 and slightly less than 1, respectively. For our as-grown CVD graphene layer shown in Fig. 2(b), the I2D/IG has a ratio greater than 4, which indicates that we have produced a high quality mono-layer on the Cu foil.

### Device Characterization

The optical transmission loss of the device was measured with a 1550 nm diode and the cut-back technique was used to remove extraneous losses. Measurements were undertaken as a function of wavelength using a low power continuous wave Tunics laser as the source and a Newport 818-IR detector. The source used for the high peak power experiments was a OneFive Origami with a bespoke pulse duration of 750 fs FWHM. The carrier dynamics were estimated by comparing the modulated response directly with the pump pulse. As the pump is symmetric, the asymmetry in the tail is attributed to the slow recovery of the carriers. The relaxation times were then determined by fitting the extended tail with a convolution of 2 exponentials.

### Numerical modelling

Numerical simulations were undertaken using the COMSOL Multiphysics with the RF module and the LiveLink to MatLab.

## Additional Information

**How to cite this article**: Zhang, H. *et al.* Enhanced all-optical modulation in a graphene-coated fibre with low insertion loss. *Sci. Rep.* **6**, 23512; doi: 10.1038/srep23512 (2016).

## Supplementary Material

Supplementary Information
