# Supplementary material for: Enhanced all-optical modulation in a graphene-coated fibre with low insertion loss
Source: Sci Rep. 2016 Mar 22;6:23512. doi: 10.1038/srep23512 (PMC4802326; doi:10.1038/srep23512)
Supplement: Supplementary Information [file srep23512-s1.pdf]

Enhanced all-optical modulation in a graphene-coated fibre with low insertion loss

Haojie Zhang[1], Noel Healy[1,2*], Li Shen[1], Chung-Che Huang[1], Dan Hewak[1] and Anna C. Peacock[1].

[1]Optoelectronics Research Centre, University of Southampton, Highfield, Southampton SO17 1BJ, UK. [2]Emerging Technology and Materials Group, School of Electrical and Electronic Engineering, Newcastle University, Newcastle upon Tyne NE1 7RU, UK.

* e-mail: noel.healy@ncl.ac.uk

Supplementary Information

SF1: Polished fibre surface characterisation

SF2: Chemical vapour deposition of graphene

SF3: Graphene transfer process

SF4: Broadband polarisation characteristics of the device

SF5: Graphene characterisation

SF1 Polished fibre surface characterisation

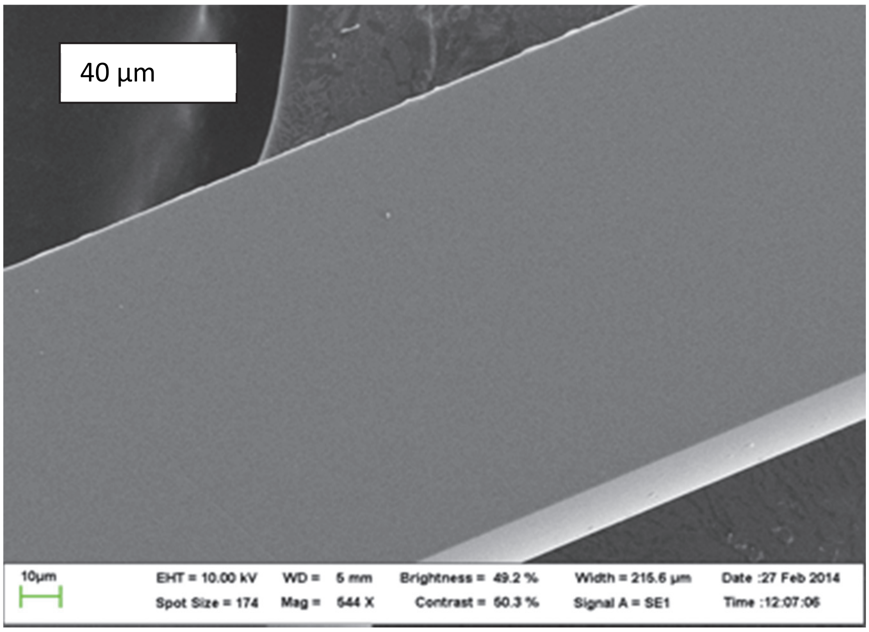

z – range 2.9 nm

y – range 34 µm

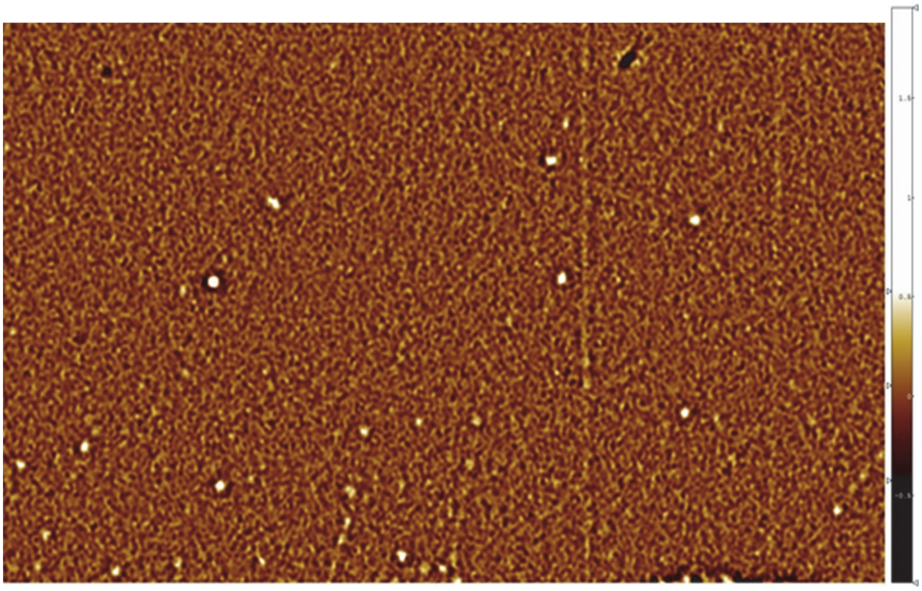

x – range 56 µm

The surface quality was measured with a ZeScope 3D optical profiler. Surface roughness: RMS = 0.8 nm, with max features ~ 1.5 nm. Flatness: a longitudinal angle of 0.04 º was measured.

SF2 Chemical vapour deposition of graphene

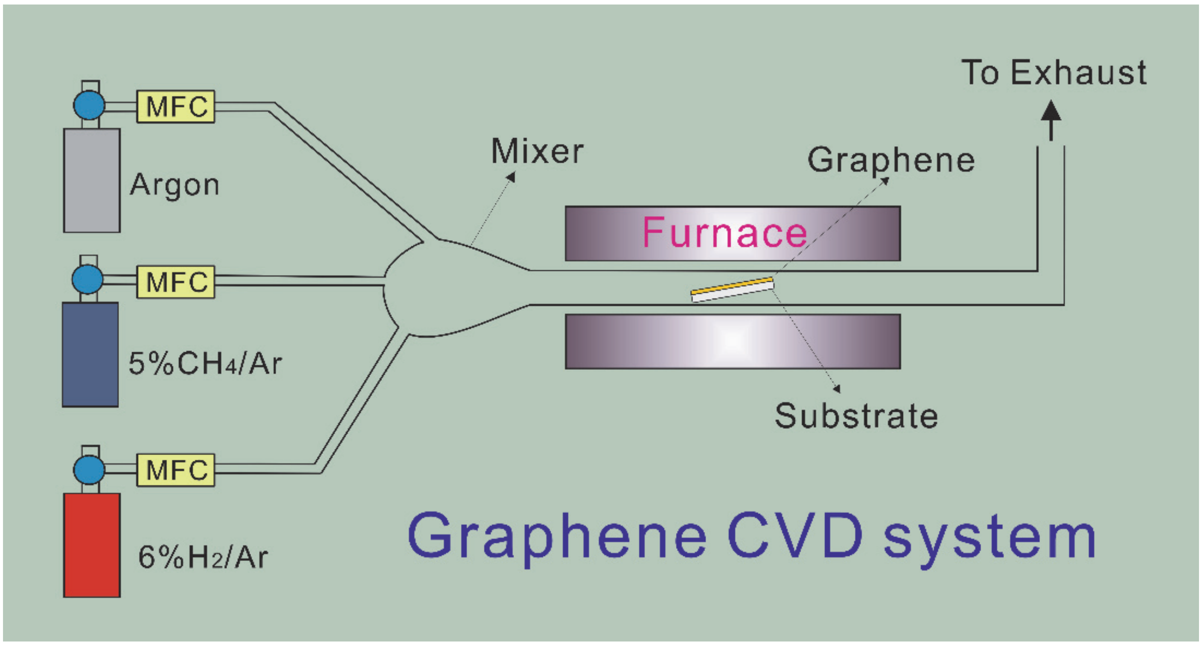

Experimental configuration of CVD growth of graphene onto a copper substrate (MFC: mass flow controller)

SF3 Process flow for graphene to fibre transfer

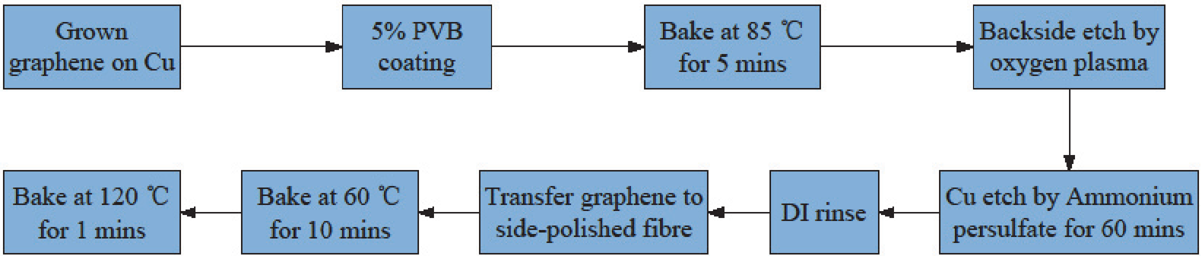

Graphene grown on copper must be transferred from the metal onto a substrate to fabricate the device. A layer of polyvinyl butyral (PVB) is deposited on top of the graphene. PVB was found to permit good contact between fibre/graphene/PVB.

SF4: Broadband polarisation characteristics of the device

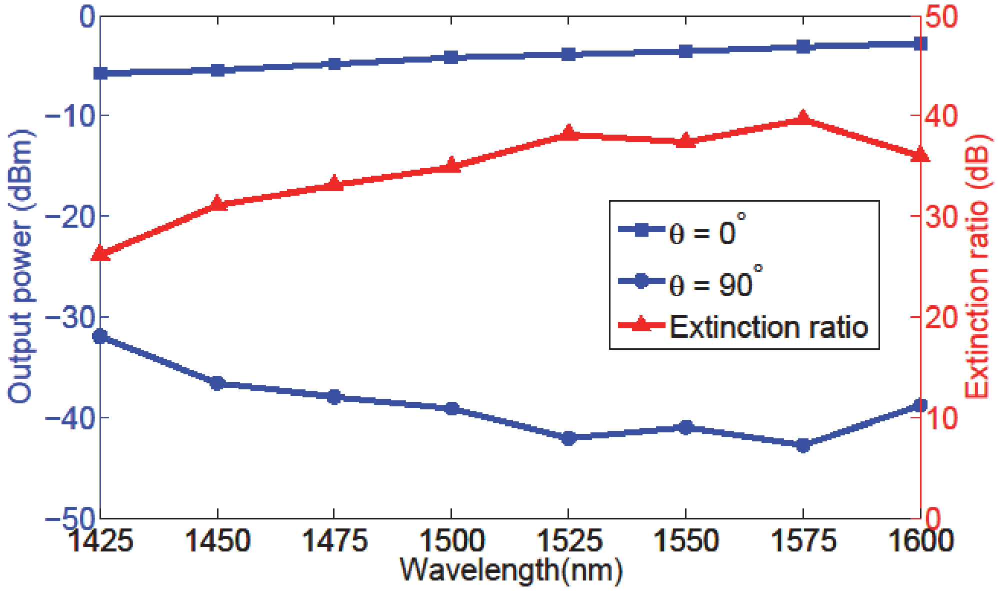

Solid line: output power at θ = 0 ° (TE) and θ = 90 ° (TM). Dashed line: corresponding extinction ratios from 1425 nm to 1600 nm. Note: connector losses included in this figure.

SF5: Graphene characterisation

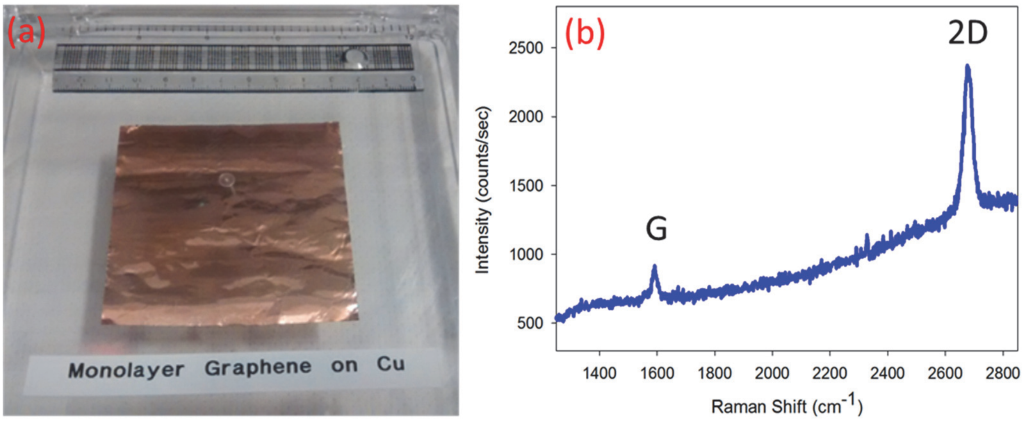

(a) A typical CVD-grown graphene on Cu foil with the size of 80 mm x 90 mm. (b) Raman spectrum of CVD-grown mono-layer graphene on Cu foil, which revealed the ratio of I_{2D} / I_G is greater than 4 and almost no D peak appeared in the spectrum.
